# Enhanced CO_2_ Capture Using TiO_2_ Nanoparticle-Functionalized Solvent: A Study on Desorption Experiments

**DOI:** 10.3390/nano15171301

**Published:** 2025-08-22

**Authors:** Mattia Micciancio, Nicola Verdone, Alice Chillè, Giorgio Vilardi

**Affiliations:** Department of Chemical Engineering Materials Environment, Sapienza University of Rome, Via Eudossiana 18, 00184 Rome, Italy; mattia.micciancio@uniroma1.it (M.M.); nicola.verdone@uniroma1.it (N.V.); alice.chille@uniroma1.it (A.C.)

**Keywords:** CO_2_ capture, reactive desorption, potassium carbonate, nanoparticles, TiO_2_

## Abstract

Cutting CO_2_ emissions is crucial to face of climate change, and one of the most tried and true means of post-combustion CO_2_ capture is by way of chemical absorption. In this work, the effect of titanium dioxide (TiO_2_) nanoparticles in a 25 wt% potassium carbonate (K_2_CO_3_) solution on solvent regeneration is investigated. This research follows the previous work in which the effect of nanofluids was evaluated on CO_2_ absorption. Desorption was studied at three different temperatures (343.15, 348.15 and 353.15 K), using the absorbent fluid with and without 0.06 wt% TiO_2_ nanoparticles. The results indicate that the nanofluid enhanced the CO_2_ release rates, also reducing energy consumption. The mass transfer was intensified by the presence of nanoparticles, which in turn increased CO_2_ diffusivity and influenced the liquid boundary layer, resulting in an enhanced desorption rate, because of the higher diffusivity. These enhancements were achieved with negligible modifications to the fluid properties, i.e., viscosity. In summary, application of TiO_2_-enhanced K_2_CO_3_ solutions is a practical approach to enhance CO_2_ removal performance and reduce operating costs such that CO_2_ capture is beginning to be environmentally and economically more competitive for the existing system retrofit.

## 1. Introduction

The growing concern over climate change has brought to the forefront the urgent need for innovative solutions to mitigate greenhouse gas emissions. The concentration of carbon dioxide in the atmosphere can be limited by using carbon capture technologies. Global warming and its associated environmental impacts are being exacerbated by the accumulation of GHGs [1], which requires strategies that can effectively curb emissions while facilitating sustainable development. Carbon capture technologies are important in the context of escalating climate concerns and international commitments to reduce emissions [2].

As the global economy expands rapidly and energy consumption rises, the world faces dual challenges: meeting its energy supply and demand while grappling with an alarming increase in CO_2_ emissions. Thus, it is crucial to implement measures to reduce greenhouse gas emissions. The Paris Agreement [3], embraced by 196 nations in 2015, emphasizes the need to decarbonize and shift to green energy, particularly focusing on the current industrial system in the short term [4].

Decarbonizing the industrial sector is daunting and costly, even with available technologies. Transitioning to a net zero-emission energy system necessitates substantial changes in energy production and usage, achievable only through a diverse array of technologies [5]. Carbon Capture, Utilization, and Storage (CCUS) technologies stand out as the only set capable of directly reducing emissions across key sectors and removing CO_2_ without fundamentally altering processes [6].

Retrofitting existing plants, especially in hard-to-abate sectors, poses a significant challenge to decarbonization efforts [7]. Typically, CCS and CCU technologies operate downstream without altering the core process, although exceptions exist such as oxycombustion or integrated calcium looping [8].

A widely recognized and effective PCC (Post Combustion Capture) technology is chemical absorption (TRL9), which generally employs solvents like monoethanolamine (MEA) to specifically extract CO_2_ from flue gases [9,10].

This work seeks to highlight the possible enhancement in the stripping/regeneration section of a classical chemical absorption process with the use of an aqueous solution of potassium carbonate (K_2_CO_3_) at 25 wt% (the absorbent used in the Benfield process [11]) as the absorbent fluid, nano-functionalized with TiO_2_ nanoparticles, working at atmospheric total pressure.

Due to its low cost, mild corrosiveness, and chemical stability, K_2_CO_3_ is well-suited for large-scale applications; it is, also, less volatile and less environmentally harmful than the classical amine-based absorbents [12]. However, due to the slower kinetics, it requires bigger devices to reach the same efficiency of other absorbents. To address this limitation, researchers have investigated a range of strategies, including the application of nanotechnology to improve solvent efficacy.

The novelty of this research is the addition of metal oxide nanoparticles to a low-cost, low-toxicity solvent known to require less regeneration energy than amines like aqueous potassium carbonate. The selection of titanium dioxide (TiO_2_) nanoparticles is supported by literature reports indicating that they exhibit superior performance in enhancing CO_2_ absorption compared to other nanomaterials, particularly without requiring the application of external magnetic fields [13].

Nanofluids, first introduced by Choi in 1995 [14] are fluids that consist of nanoparticles dispersed in a base liquid. Nanofluids provide enhanced thermal characteristics and surface area, both of which can increase the absorption and release of CO_2_ in the capture solvents. Such enhanced characteristics support greater gas separation effectiveness. Nanofluids further assist in controlling the temperature oscillations in the system through enhanced heat transfer, thereby ensuring enhanced absorption and regeneration performance and reducing the energy necessary for regeneration, ultimately reducing the process costs for carbon capture [15,16].

The precise mechanism responsible for the enhanced CO_2_ absorption by nanofluids remains uncertain. Nonetheless, several studies suggest that this improvement likely arises from a combination of multiple interacting mechanisms rather than a single dominant factor [17]. These effects can be summarized in three different categories:

- Activation energy effect: the presence of nanoparticles (NPs) leads to more frequent collisions between liquid molecules and particles and also raises the average activation energy of particles in the solution [18].
- Surface effect: the introduction of nanoparticles alters the boiling surface characteristics, including nucleation site density, heat transfer area and surface roughness [19]. Due to the high temperatures in the regeneration section, nanoparticle deposition on the heater surface becomes more pronounced, leading to changes in the surface topology of the electrical coil. This results in the formation of a porous layer with greater wettability and roughness compared to the original, untreated heater surface [20].
- Thermal conductivity enhancement, nanoparticles possess significantly higher thermal conductivity than the base fluid, so incorporating them into the liquid phase increases the overall thermal conductivity of the solvent. As a result, nanofluids enable faster gas stripping due to more efficient energy dispersion and quicker temperature rise [21,22,23].

In this context, the present study explores the impact of TiO_2_ nanoparticles on improving the regeneration performance of aqueous potassium carbonate (K_2_CO_3_) solutions used in post-combustion CO_2_ capture. By integrating experimental desorption data with a kinetic evaluation of mass transfer and reaction rates, this work aims to demonstrate the potential of nanofluid-enhanced solvents to increase CO_2_ desorption efficiency. The findings contribute to the development of more energy-efficient and environmentally sustainable CCS processes, in line with industrial decarbonization goals and global climate commitments.

## 2. Materials and Methods

### 2.1. Materials

The reagents potassium carbonate (ACS reagent 99.0%) and titanium (IV) oxide-nanopowder (99.9%, anatase phase, ~25 nm particle size) were supplied by Sigma Aldrich^®^ (St. Louis, MO, USA) and were used as received. Gaseous carbon dioxide and nitrogen were purchased at technical grade.

### 2.2. Experimental Apparatus

**Figure 1:**
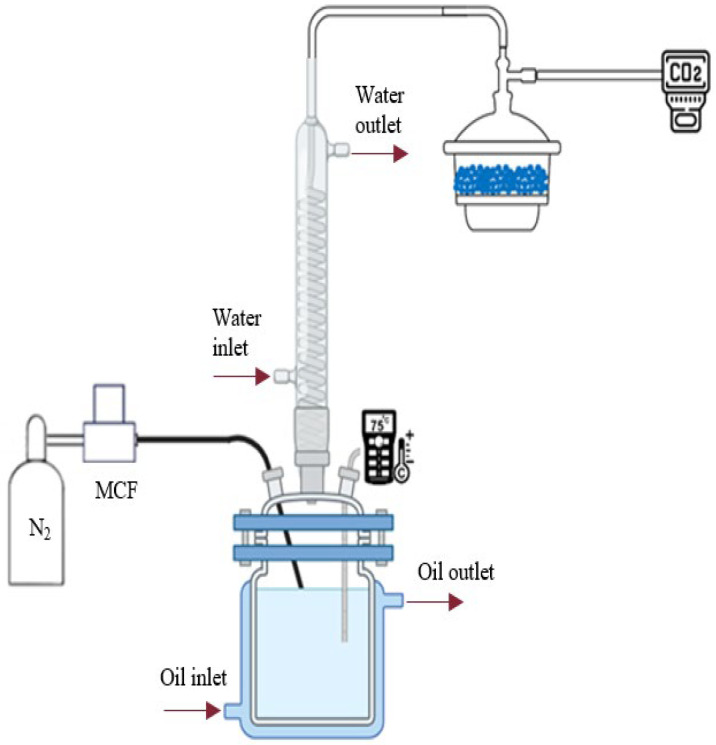
Experimental apparatus for CO_2_ desorption tests: (1) nitrogen cylinder, (2) mass flow controller, (3) jacketed stirred cell reactor, (4) thermocouple, (5) bubble condenser, (6) silica gel dryer, (7) IR analyzer (DAQ: data acquisition unit).

Desorption tests were conducted in a stirred cell reactor unit. The schematic of the experiment is depicted in Figure 1. Technical-grade nitrogen (N_2_) was employed as an inert carrier gas to assist in the removal of CO_2_ from the solvent during the desorption phase. In this context, N_2_ is not a target component but serves as a medium to promote desorption. Additionally, the bubbling of N_2_ through the liquid generates local turbulence, which enhances mass transfer in the liquid phase.

The N_2_ gas flow rate was accurately controlled using a mass flow controller (MFC) by a valve directing the gas into the vessel where the CO_2_-rich absorbent solution was placed. The nitrogen gas flow rate was maintained at 900 mL/min for all experiments to ensure consistent conditions across tests.

#### 2.2.1. Absorbent Preparation and Reactor Setup

The base absorbent was a 100 mL solution of 25 wt% K_2_CO_3_. To prepare the nano-absorbent, TiO_2_ NPs were dispersed in the base fluid. In order to get a well-dispersed nanofluid and overcome nanoparticles gathering, a 20 min ultrasonic treatment under the frequency of 52 kHz was applied to the nanofluid before each absorption and desorption.

Ultrasonic treatment was applied in the laboratory setting to ensure a reproducible dispersion of the nanoparticles; without stabilizers or careful control (e.g., continuous mixing, pH control), TiO_2_ suspensions tend to slowly settle out over time. Empirical data indicate that ionic strength and pH strongly influence stability: increasing salt or adjusting pH near the TiO_2_ point-of-zero charge greatly accelerates aggregation [24]. In real absorbent streams (which may have high ionic strength or reactive solutes), even ultrasonic dispersion may be insufficient to ensure stability for weeks or months without additives.

TiO_2_ can be recycled by simple decantation or centrifugation. For instance, gravity settling and decanting recovered ~77% of commercial TiO_2_ photocatalyst for reuse [25]. Many studies of TiO_2_ photocatalysis confirm that recycled nanoparticles retain their activity for ≥5–10 cycles [26].

The nanoparticle concentration used (0.06 wt%) corresponded to the optimal value identified in previous CO_2_ absorption tests [13]. The loading of the rich-absorbent fluid changed between the case with nanoparticles and without; in the scenario without the nanoparticles the rich-loading ranged from 0.092 to 0.101 $mol_{CO_{2}}/mol_{K_{2}CO_{3}}$, and in the scenario with nanoparticles the rich-loading of the absorbent fluid ranged from 0.134 to 0.142 $mol_{CO_{2}}/mol_{K_{2}CO_{3}}$.

The nanoparticles do not directly participate in the chemical reaction, but they enhance diffusivity and thus increase the loading capacity over the same time span. The increase in the absorption capacity reported is aligned with the outcome that are also reported in the study of Rahmatmand [27], Aghel [28] and Haghtalab [29]; this is likely based on an effect resulting from both absorption in the base fluid and a physical adsorption on the nanoparticulate matter of the nanoparticles.

The temperature at which the absorption part of the tests was performed is 70 °C. A hermetically sealed lid on the vessel for the absorbent defined inlets for gas feed and outlet, and the whole apparatus was placed on a magnetic stirrer (AREX 5 digital; Velp Scientifica Srl, Usmate, Italy) set to 300 rpm to provide good mixing of the liquid phase. An external jacket, which is connected to a thermostatic bath, CORIO CD-200F (Julabo, Seelbach, Germany), was also inserted for temperature stabilization.

The temperature of the service fluid was varied between 95 and 115 °C. Thermal H10 oil (operating range: −40 to 180 °C) was employed as the service fluid. However, the process fluid reached lower temperatures (70–80 °C), as reported in Table 1. This discrepancy is attributed to the thermal resistance of the reactor wall and the specific configuration of the thermostatic bath.

#### 2.2.2. Experimental Procedure and Gas Analysis

The system under examination was equipped with a bubble condenser and a silica gel dryer to dry the gas stream immediately before analysis with an infrared analyzer (model 4400 IR ADEV, Cesano Maderno, Italy, measurement range 0–40% and a resolution of 0.01 vol% CO_2_) ensuring consistent and reliable analytical results. Recovered water was condensed and reintegrated in the vessel, thereby reducing solvent losses during tests. Through the IR analyzer it was possible to follow in real time the quantity of CO_2_ released by the liquid, which yielded information about the desorption kinetics and the efficiency of the different systems surveyed. The liquid phase was treated in batch mode, and the nitrogen phase in continuous mode to keep turbulence in the system. The system was purged with nitrogen gas to evacuate residual air and CO_2_ prior to each measurement. It was confirmed with continuous monitoring that such levels were undetectable. Desorption efficiency was assessed by online monitoring of the released CO_2_ in the gas outlet stream using the IR analyzer. The CO_2_ concentration in the outflow was recorded in real time using the IR analyzer to compile a time profile of CO_2_ levels. This enabled us to dynamically follow the desorption process and determine when the test was finished.

In each test the CO_2_ concentration, measured by the IR analyzer, increased during the initial period of the test (this rise was evidence of the increase of desorption of CO_2_ with the absorbent temperature that was followed by a thermocouple); after few minutes no further increase in the CO_2_ concentration occurred, having reached a maximum, from which a decrease began. This behavior implies the slowing of the kinetics with the reduction in the quantity of CO_2_ inside the absorbent. To verify the improvement in desorption efficiency, experiments were performed in an initial reference system (absorbent: a basic solution of K_2_CO_3_) and in the reference solution after the addition of little content of TiO_2_ nanoparticles.

#### 2.2.3. Absorbent Temperature

During the various desorption tests, the temperature of the absorbent fluid was measured using a thermocouple (type K thermocouple, QUARKZMAN, Tianhe Guangzhou, China, measurement range from 0 to 600 °C), stabilized after about 15 min at values lower than those of the service fluid, due to heat losses through the reactor wall. The temperatures reached are those, respectively, in Table 1:

This difference in temperature, between the service fluid and the process fluid, is due to the jacket used to heat the process fluid itself.

The temperatures here reported are measured on the rich-absorbent that was subjected at a batch absorption process with a simulated flue gas composed of 15% of CO_2_ and 85% of N_2_.

### 2.3. Assessment of CO_2_ Desorption Rate

The quantity of CO_2_ desorbed from the absorbent fluid was extracted by analyzing the data obtained from the IR analyzer. The CO_2_ desorption rate was determined considering the following assumptions: (i) CO_2_ behaves as an ideal gas, (ii) the mass transfer resistance in the gas phase is negligible, (iii) the gas–liquid interfacial area coincides with the superficial area of the nitrogen bubble that cross the absorbent liquid in the reactor tank. Assuming the above assumptions are valid, the molar flux of CO_2_ desorbed from the solution ($N_{CO_{2}}$ [mol s^−1^]) can be calculated by the following expression [30]:

(1) $$N_{CO_{2}}=K_{L}A\left(C_{CO_{2}}^{*}-\;C_{CO_{2}}\right)$$

where $C_{CO_{2}}^{*}$ [mol/m^3^] and $C_{CO_{2}}$ [mol/m^3^] are the CO_2_ concentration at the liquid–gas interfaces and in the bulk of the liquid phase, A [m^2^] is the gas–liquid interfacial area and $K_{L}$ is the global mass transfer coefficient. $K_{L}$ can be described as the inverse of the sum of the local mass transfer coefficients in the liquid ($k_{L}$) and the gas phase ($k_{G}$), the second, modified using Henry’s law constant for the CO_2_ ($H_{CO_{2}}$ [P atm^3^ mol^−1^]) and the enhancement factor (E), as mentioned in Equation (2).

(2) $$\frac{1}{K_{L}}=\frac{1}{k_{L}}+\frac{E}{H_{CO_{2}}\;k_{G}}$$

The global coefficient expression can be further simplified, because the local mass transfer coefficient in the gas phase is negligible compared to the liquid one, so the Equation (2) becomes as follows:

(3) $$K_{L}=k_{L}$$

To quantify the variation in the results between the tests with the nanoparticles and without the nanoparticles, we considered that the whole increase in the desorption kinetics is due to the increase in the diffusivity of the gas, to evaluate this change we used Equation (4), that is used to evaluate the Hatta number (Ha) [30]:

(4) $$Ha=\frac{\sqrt{D_{CO_{2}}\;k_{ov}}}{k_{L}}$$

where the $D_{CO_{2}}$ [m^2^ s^−1^] is the diffusivity of the CO_2_, and k_ov_ [s^−1^] is the overall reaction constant.

Using the Equations (1), (3) and (4) we obtained the Equation (5) from which we have a way to evaluate the $K_{L}$ value.

(5) $$K_{L}=\frac{N_{CO_{2}}}{\;A\left(C_{CO_{2}}^{*}-C_{CO_{2}}\right)}=\frac{\sqrt{D_{CO_{2}}\;k_{ov}}}{Ha}$$

Using Equation (5) and by multiplying both sides of the equation by the factor $\frac{V}{RT}$, the flux $N_{CO_{2}}$ [Pa s^−1^] can be reformulated as follows:

(6) $$N_{CO_{2}}=\;\frac{\sqrt{D_{CO_{2}}\;k_{ov}}}{Ha}\;\frac{V}{RT}\;A\;\left(C_{CO_{2}}^{*}-C_{CO_{2}}\right)=m\;∆C_{CO_{2}}$$

where m [s^−1^] is the slope of the curve obtained plotting the experimental data for each test $N_{CO_{2}\;}vs.\;{∆C}_{CO_{2}}$.

## 3. Results and Discussion

### 3.1. Ionization Constants

Accurate determination of kinetic parameters for carbon dioxide reactions in potassium carbonate solutions requiring the evaluation of the stoichiometric constants for the ionization of carbonic acid (K_1_) and bicarbonate ion (K_2_) into K_2_CO_3_ solutions were based on temperature dependency, as described by Equations (7) and (8) [31]. The results obtained from the Equations (7) and (8) are reported in Table 2.

(7) $$\log\left(K_{1}\right)=-\frac{3404.7}{T}+14.843-0.03279T$$

(8) $$\log\left(K_{2}\right)=-\frac{2902.4}{T}+6.498-0.0238T$$

### 3.2. Kinetic Constants

The rate constant, $k_{H_{2}O}$, for the hydration reaction of CO_2_ was regarded as a temperature-related parameter and estimated using the empirical correlation, suggested by Peirce et al. [31], given in Equation (9).

(9) $$log(k_{H_{2}O})=329.85-110.541\log\left(T\right)-\frac{17,265.4}{T}$$

Moreover, the hydroxylation rate constant of CO_2_ (*k_OH_*) in aqueous potassium carbonate solutions was calculated as a function of temperature and ionic strength (*I*) in accordance with the relationships proposed by X. Ye and Y. Lu [32] using the following Equations (10)–(13). These correlations are applicable in the range of temperature between 25–80 °C, ionic strength of up to 12 kmol·m^−3^.

(10) $$log(k_{OH})=ln(A)-\frac{E_{a}}{RT}$$

(11) $$E_{a}=47.03$$

(12) $$\log\left(A\right)=0.24I+26.4$$

(13) $$I=\frac{1}{2}\sum_{i=1}^{N}C_{i}z_{i}^{2}$$

where *C_i_* is the concentration of the *i*-th ionic species and *z_i_* its charge. The kinetic constants were obtained by fitting the experimental data with the reaction-absorption principles and summarized in Table 3.

The results reveal that the contribution of hydration to the total reaction kinetics is small, so that the hydroxylation reaction step takes more part in CO_2_ conversion when pH is higher than 8. In this way, the kinetic model can be expressed as the following simplified cases according to Equation (14).

(14) $$k_{OV}=k_{H_{2}O}+k_{OH^{-}}\cdot \left[OH^{-}\right]\;\to \;k_{OV}=k_{OH^{-}}\cdot \left[OH^{-}\right]$$

As indicated above, the kinetic of the hydroxylation reaction can be expressed in a pseudo-first-order fashion.

This approximation is well justified by the assumption that K_2_CO_3_ forms a buffer solution when dissolved in water, further stabilizing the pH and supporting the assumption of a constant hydroxide ion concentration throughout the reaction.

### 3.3. Desorption Without Nanoparticles

The initial tests were conducted on a reference system, in particular this system was constituted of the base fluid, consisting of a 100 mL of 25 wt% aqueous solution of K_2_CO_3_ as the absorbent. The desorption tests were conducted, as already said, on the absorption samples produced in the best condition, to guarantees the highest CO_2_ absorption; so, the absorption tests were conducted at 70 °C.

**Figure 2:**
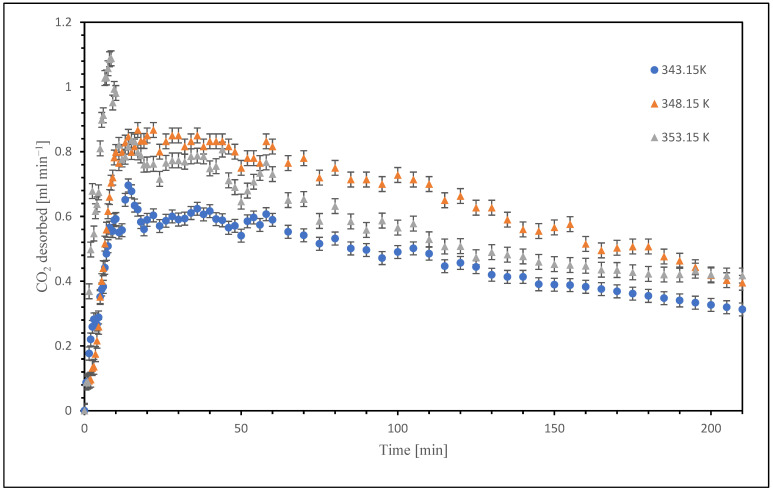
Trend of CO_2_ desorbed in the base fluid.

The desorption tests were conducted at three different process fluid temperatures, which are: 343.15 K, 348.15 K and 353.15 K. The different results obtained are plotted in Figure 2.

As can be seen, the peak of CO_2_ desorption rises with the increase of the temperature, this is due to the fact that both the thermodynamics and the kinetics are favored by the increase of the temperature.

As shown in Figure 2, the test conducted at the highest temperature (353.15 K) resulted in the highest peak of CO_2_ desorption rate. However, the desorbed CO_2_ amount subsequently decreased over time and fell below that observed at 348.15 K.

**Figure 3:**
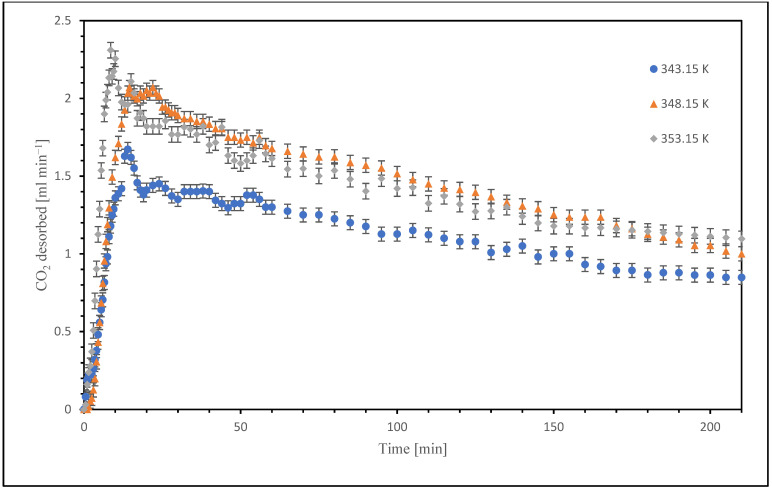
Trend of CO_2_ desorbed in the nanofluid.

The error bars reported in Figure 2 and Figure 3 are obtained from the standard deviation of replicants.

### 3.4. Desorption with Nanoparticles

Subsequent tests were carried out using the same conditions as in the previous tests, i.e., 100 mL of 25 wt% aqueous solution of K_2_CO_3_ as the base case, and then functionalizing it with 0.06 wt% TiO_2_ nanoparticles. The choice of the number of nanoparticles to be used was due, as with the choice of absorption temperature, to reproduce the best conditions for achieving maximum CO_2_ absorption by the absorbent fluid [15].

After absorption tests were performed on the nano-functionalized samples, they were subjected to desorption at 3 different temperatures, as in the base case. The results obtained from these tests have similar trends to those obtained from the blank tests (tests without nanoparticles), the difference being in the different kinetics of desorption and the increase in the maximum amount of CO_2_; in fact, as can be seen from Figure 3, similar curves were obtained, but they have a steeper initial section, going to indicate a faster CO_2_ desorption rate.

The addition of nanometer-sized TiO_2_ powders in the absorbent fluid significantly enhanced the desorption performance of the absorbent solution. In particular, the tests carried out with 0.06 wt% TiO_2_, which was the concentration optimized from the absorption campaign, showed improved CO_2_ release after CO_2_ sequestration relative to the base fluid itself. This is mainly due to the increment in the value of the overall mass diffusivity of CO_2_ in the liquid phase.

The increase in diffusivity is correlated with the increase of the Schmidt number and the increased frequency of molecular impacts within the fluid caused by the dynamics of the nanoparticles.

In addition, the random movement of the nanoparticle according to Brownian motion is thought to disturb the boundary layer at the gas bubbles, which in turn causes a reduction of liquid film thickness and then enhances the gaseous-liquid mass transfer.

These effects collectively contribute to a steeper initial slope in the CO_2_ desorption curves indicating faster desorption kinetics when compared to the non-functionalized fluid.

However, it should also be noted that the inclusion of nanoparticles results in a slight increase in fluid viscosity and density. Although these changes can potentially hinder mass transfer, the effect remains negligible at the tested concentration of 0.06 wt%. It is only at higher volume fractions—typically above 2% [33]—that these physical property changes might significantly reduce desorption efficiency.

**Figure 4:**
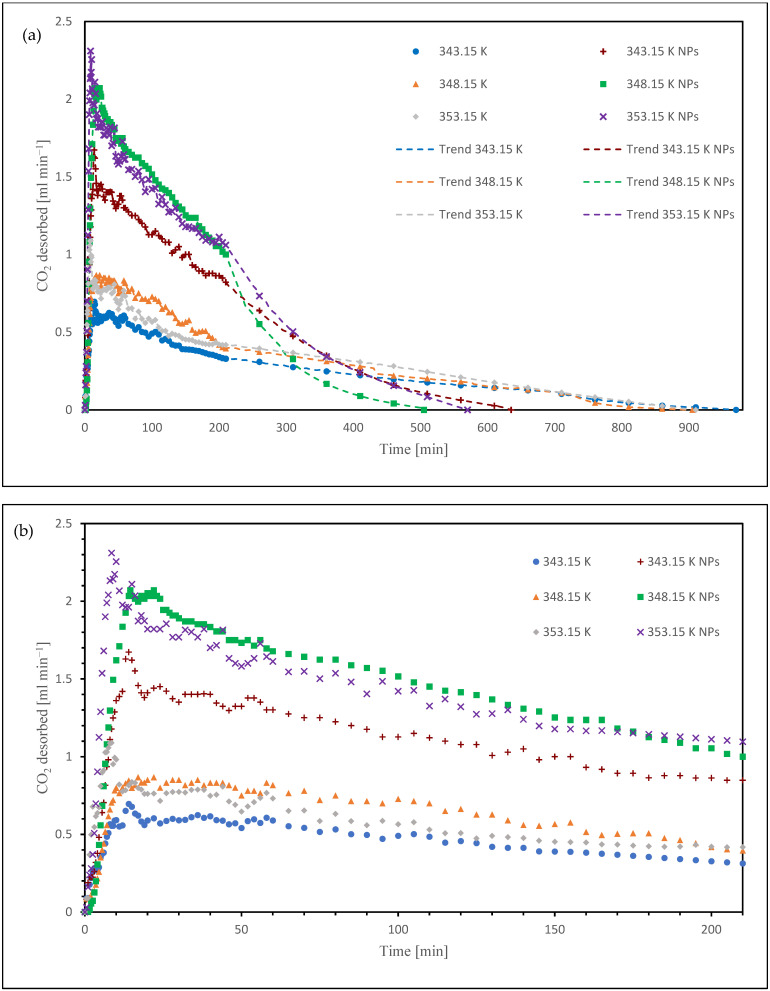
(**a**) Comparison of the trend of CO_2_ desorbed in the nanofluid until complete desorption; (**b**) Comparison of the trend of CO_2_ desorbed in the nanofluid until 210 min.

Figure 4a,b show the difference between the desorption trend between the tests without and with the nanoparticles (NPs). Figure 4a also shows the trends of the experimental data points. This behavior arises from the fact that, after 210 min, measurements were recorded at 50 min intervals until desorption was complete.

The total quantity of CO_2_ desorbed corresponds to the amount initially absorbed, as the K_2_CO_3_ solution undergoes near-complete regeneration in batch set-up, with only negligible residual loading. Consequently, the final CO_2_ loading after desorption is effectively zero [34,35].

### 3.5. Kinetics Results and Evaluation of CO_2_ Diffusion Coefficient

Based on Equation (6) and the laboratory data, the slope coefficient (m) for each test was determined by plotting the flow rate $N_{CO_{2}}$ as a function of the desorbed $∆C_{CO_{2}}$. It was possible to determine the rate constant using Equation (15):

(15) $$m=\;\frac{\sqrt{D_{CO_{2}}\;k_{ov}}}{Ha}\;\frac{V}{RT}\;A$$

The coefficient of m was evaluated considering the first section of the curve, the linear one. In deriving Equation (6), the CO_2_ concentration at the gas–liquid interface is assumed to be negligible compared to the bulk liquid-phase concentration, thereby simplifying the calculation of $\Delta C_{CO_{2}}$, that was then correlated to the CO_2_ exiting the system that was measured by the IR analyzer.

To investigate the role of nanoparticles, the rate constant ($k_{ov}$) was first determined (Equation (14)) and then kept fixed while evaluating CO_2_ diffusivity in the presence of nanoparticles. The value of A was evaluated as it was in the previous study [13]. This method provided a clearer understanding of how nanoparticles affect the diffusion behavior of CO_2_ within the system. As shown in Table 4, the diffusivity of CO_2_ increases when nanoparticles are introduced, compared to the baseline scenario without them.

The presence of nanoparticles appears to enhance mass transfer efficiency by promoting a shuttle-like mechanism and thinning the boundary layer of the liquid. These findings are backed by experimental data, which indicate a quicker absorption rate relative to that of the pure solvent

### 3.6. Evaluation of Desorption Duty

Other than the kinetics, the electrical power used to strip the CO_2_ from the absorption fluid was also estimated. The power measured was the one needed to reheat the service fluid used to maintain the temperature of the absorption fluid constant.

To measure the power provided to heat the service fluid, a wattmeter was used connected to the thermostatic bath.

From the results obtained it was possible to estimate the percentage reduction of electrical consumption over the mass of CO_2_ desorbed $\left[kW\cdot kg_{CO_{2}}^{-1}\right]$. As is highlighted in other literature studies, the addition of the nanoparticles to the absorbent fluid reduces the duty requirement at the reboiler, needed to strip the acid gas from the rich-solvent [15].

In Table 5, the percentage reduction of electrical power used between the fluid with and without nanoparticles at the different temperatures are reported.

The estimates reported in the table are based on the measurement of the electrical power used by the thermostatic bath, that considers both the duty needed to heat the service fluid and also the electrical power absorbed by the pump integrated in the bath itself, so the value estimated can be used as indication of the real difference. As a matter of fact, this comparison is only a first indication of the possible energy consumption reduction because of the use of NPs, but further studies are necessary on a higher scale (and with continuous experimental configuration) to perform a more accurate quantification of this effect.

## 4. Conclusions

In this work, the regeneration performance of potassium carbonate-based absorbent solutions functionalized with a low concentration of TiO_2_ nanoparticles was investigated for post-combustion CO_2_ capture applications. Desorption experiments conducted at three different temperatures (343.15, 348.15 and 353.15 K) demonstrated that the addition of 0.06 wt% of TiO_2_ nanoparticles significantly improves desorption kinetics and CO_2_ diffusion rates, without causing adverse effects on fluid properties such as viscosity or density at the tested concentration.

The presence of nanoparticles led to an increase in the ratio $\sqrt{D_{CO_{2}}}/Ha$ of up to 62% at 343.15 K compared to the base case, rising from 4.905 to 7.963 × 10^8^ $\left[m\cdot s^{-\frac{1}{2}}\right]$. The initial desorption rate was consistently higher in nanofluid systems, with sharper CO_2_ desorption curves and shorter plateau times, indicating enhanced mass transfer dynamics and more efficient regeneration performance.

Additionally, experimental results demonstrated that nanofluid-enhanced solvents are not only characterized by a larger rate of CO_2_ desorption but also by reduced energy consumption associated with solvent regeneration, confirming that nanotechnology provides a promising pathway to enhance the overall efficiency and cost-effectiveness of chemical absorption-based carbon capture systems.

The observed improvements are attributed to multiple phenomena, including increased thermal conductivity, enhanced mass diffusivity and disturbance of the boundary layer due to Brownian motion of the nanoparticles. These effects collectively reduce resistance to mass transfer at the gas–liquid interface, resulting in faster desorption kinetics and more favorable thermodynamic conditions.

Overall, the findings suggest that the application of TiO_2_ nanofluids in aqueous K_2_CO_3_ solutions represents a viable strategy for improving the performance of PCC systems, particularly in terms of desorption efficiency and energy demand. This aligns well with industrial decarbonization efforts and international climate change mitigation goals.

However, it is important to note that this study employed a baseline absorbent (K_2_CO_3_) with a relatively low intrinsic desorption capacity, in particular at atmospheric pressure. Future research should explore alternative absorbents with higher reaction kinetics and optimize nanoparticle design. Moreover, advanced kinetic modeling and dynamic simulations will be essential to accurately interpret nanoparticle–solvent–CO_2_ interactions, while pilot-scale studies should aim to validate the observed benefits under continuous operating conditions and assess long-term stability and techno-economic feasibility.

## Figures and Tables

**Table 1:** Variation of the temperature of the process fluid with the temperature of the service fluid.

Service Fluid Temperature (°C)	Process Fluid Temperature (°C)
95	70
105	75
115	80

**Table 2:** Stoichiometric constants for the ionization of carbonic acid (K_1_) and bicarbonate ion (K_2_) in K_2_CO_3_ solutions.

T [K]	Log(K_1_)	K_1_	Log(K_2_)	K_2_
343.15	−6.331	4.669 × 10^−7^	−10.127	7.463 × 10^−11^
348.15	−6.352	4.443 × 10^−7^	−10.125	7.506 × 10^−11^
353.15	−6.378	4.191 × 10^−7^	−10.126	7.489 × 10^−11^

**Table 3:** Rate constants of *k_OH_* and $k_{H_{2}O}$.

T [K]	*K_OH_* [m^3^ kmol^−1^ s^−1^]	$K_{H_{2}O}$ [s^−1^]
343.15	142,962.742	0.182
348.15	181,125.101	0.194
353.15	227,988.748	0.202

**Table 4:** Calculation of CO_2_ diffusivity.

Nanofluid [wt%]	T [K]	$\sqrt{D_{CO_{2}}}/Ha(\times 10^{8})\left[m\cdot s^{-\frac{1}{2}}\right]$
0	343.15	4.905
	348.15	5.167
	353.15	7.204
0.06	343.15	7.963
	348.15	9.061
	353.15	9.461

**Table 5:** Quantification of the reduction of electrical power used.

T [K]	Percentage Reduction
343.15	57%
348.15	50%
353.15	54%

## Data Availability

The data presented in this study are available on request from the corresponding author.
